# Supplementary material for: Intervertebral Disc Disease of the Lumbar Spine in Health Personnel with Occupational Exposure to Patient Handling—A Systematic Literature Review and Meta-Analysis
Source: Int J Environ Res Public Health. 2020 Jul 4;17(13):4832. doi: 10.3390/ijerph17134832 (PMC7370072; doi:10.3390/ijerph17134832)
Supplement: Supplementary file 1 [file ijerph-17-04832-s001.zip › Supplementary file_4.pdf]

Supplementary file 4

Table S9. Detailed judgment for risk of bias assessments.

**Author, year**	**Bias**	**Author's judgment**	**Support for judgment**
Heliovaara, 1987	**Random sequence generation (selection bias)**	High risk	No randomisation possible due to research question.
	**Allocation concealment (selection bias)**	High risk	Allocation was made by the professional title and could therefore not be concealed.
	**Blinding of participants and personnel (performance bias)**	High risk	Exposure impossible to blind.
	**Blinding of outcome assessment (detection bias)**	Unclear risk	Outcome was adopted from existing ICD codes.
	**Incomplete outcome data (attrition bias)**	Low risk	No losses to follow-up.
	**Selective reporting (reporting bias)**	Low risk	Pre-specified outcome was reported.

**Author, year**	**Bias**	**Author's judgment**	**Support for judgment**
Hartwig et al., 1997	**Random sequence generation (selection bias)**	High risk	No randomisation possible due to research question.
	**Allocation concealment (selection bias)**	High risk	Allocation was made by the professional title and could therefore not be concealed.
	**Blinding of participants and personnel (performance bias)**	High risk	Exposure impossible to blind.
	**Blinding of outcome assessment (detection bias)**	Unclear risk	No information provided.
	**Incomplete outcome data (attrition bias)**	Low risk	Cross sectional analysis.
	**Selective reporting (reporting bias)**	Low risk	Pre-specified outcome was reported.

**Author, year**	**Bias**	**Author's judgment**	**Support for judgment**
Savage et al., 1997	**Random sequence generation (selection bias)**	High risk	No randomisation possible due to research question.
	**Allocation concealment (selection bias)**	High risk	Allocation was made by the professional title and could therefore not be concealed.
	**Blinding of participants and personnel (performance bias)**	High risk	Exposure impossible to blind.
	**Blinding of outcome assessment (detection bias)**	Unclear risk	Scans were independently assessed by two radiologists. No further information was provided.
	**Incomplete outcome data (attrition bias)**	High risk	89 (60%) of the original study population. Losses likely to affect the final results.
	**Selective reporting (reporting bias)**	Low risk	Pre-specified outcome was reported.

Table Sx. (continued)

Author, year	Bias	Author's judgment	Support for judgment
Michaelis et al., 2002	**Random sequence generation (selection bias)**	High risk	No randomisation possible due to research question.
	**Allocation concealment (selection bias)**	High risk	Allocation was made by the professional title and could therefore not be concealed.
	**Blinding of participants and personnel (performance bias)**	High risk	Exposure impossible to blind.
	**Blinding of outcome assessment (detection bias)**	Unclear Risk	No information provided.
	**Incomplete outcome data (attrition bias)**	Low risk	Case-control analysis.
	**Selective reporting (reporting bias)**	Low risk	Pre-specified outcome was reported.

Author, year	Bias	Author's judgment	Support for judgment
Chung et al., 2013	**Random sequence generation (selection bias)**	High risk	No randomisation possible due to research question.
	**Allocation concealment (selection bias)**	High risk	Allocation was made by the professional title and could therefore not be concealed.
	**Blinding of participants and personnel (performance bias)**	High risk	Exposure impossible to blind.
	**Blinding of outcome assessment (detection bias)**	Unclear risk	Outcome was adopted from existing ICD codes.
	**Incomplete outcome data (attrition bias)**	Low risk	National Health Insurance (NHI) Research Database. No losses.
	**Selective reporting (reporting bias)**	Low risk	Pre-specified outcome was reported.

Author, year	Bias	Author's judgment	Support for judgment
D'Agostin & Negro, 2017	**Random sequence generation (selection bias)**	High risk	No randomisation possible due to research question.
	**Allocation concealment (selection bias)**	High risk	Allocation was made by the professional title and could therefore not be concealed.
	**Blinding of participants and personnel (performance bias)**	High risk	Exposure impossible to blind.
	**Blinding of outcome assessment (detection bias)**	Unclear risk	Outcome was assessed on the basis of radiological reports. No further information was provided.
	**Incomplete outcome data (attrition bias)**	Low risk	Cross sectional analysis.
	**Selective reporting (reporting bias)**	Low risk	Pre-specified outcome was reported.

Table Sx. (continued).

Author, year	Bias	Author's judgment	Support for judgment
Makino et al., 2017	**Random sequence generation (selection bias)**	High risk	No randomisation possible due to research question.
	**Allocation concealment (selection bias)**	High risk	Allocation was made by the professional title and could therefore not be concealed.
	**Blinding of participants and personnel (performance bias)**	High risk	Exposure impossible to blind.
	**Blinding of outcome assessment (detection bias)**	Unclear risk	Scans were independently assessed by three spine surgeons. No further information was provided.
	**Incomplete outcome data (attrition bias)**	High risk	261 (75%) participants lost to follow-up. Reasons for the losses were non-response or refusal to participate. Losses likely to affect the final results.
	**Selective reporting (reporting bias)**	Low risk	Pre-specified outcome was reported
