# Supplementary material for: Intervertebral Disc Disease of the Lumbar Spine in Health Personnel with Occupational Exposure to Patient Handling—A Systematic Literature Review and Meta-Analysis
Source: Int J Environ Res Public Health. 2020 Jul 4;17(13):4832. doi: 10.3390/ijerph17134832 (PMC7370072; doi:10.3390/ijerph17134832)
Supplement: Supplementary file 1 [file ijerph-17-04832-s001.zip › Supplementary file_5.pdf]

Supplementary file 4

Table S10. Sensitivity analysis of studies included in the quantitative analysis (meta-analysis)

Excluded study/group	Tau²	Chi²; df [p-value]	I²	OR [95%CI]
--	0.15	6.60; 4 [0.16]	39%	2.45 [1.41, 4.26]
Heliovaara 1987 †	0.27	6.27, 3 [0.10]	52%	2.72 [1.34, 5.55]
Savage 1997 ¶	0.00	2.70, 3 [0.44]	0%	3.02 [1.84, 4.95]
Hartwig 1997 †	0.07	3.98, 3 [0.26]	25%	2.13 [1.28, 3.53]
Michaelis 2001 ‡	0.24	5.91, 3 [0.12]	49%	2.31 [1.15, 4.63]
D'Agostin 2017 †	0.24	5.91, 3 [0.12]	49%	2.32 [1.15, 4.65]

† Nurses; ‡ Nurses & geriatric nurses; ¶ Hospital porters & ambulance men

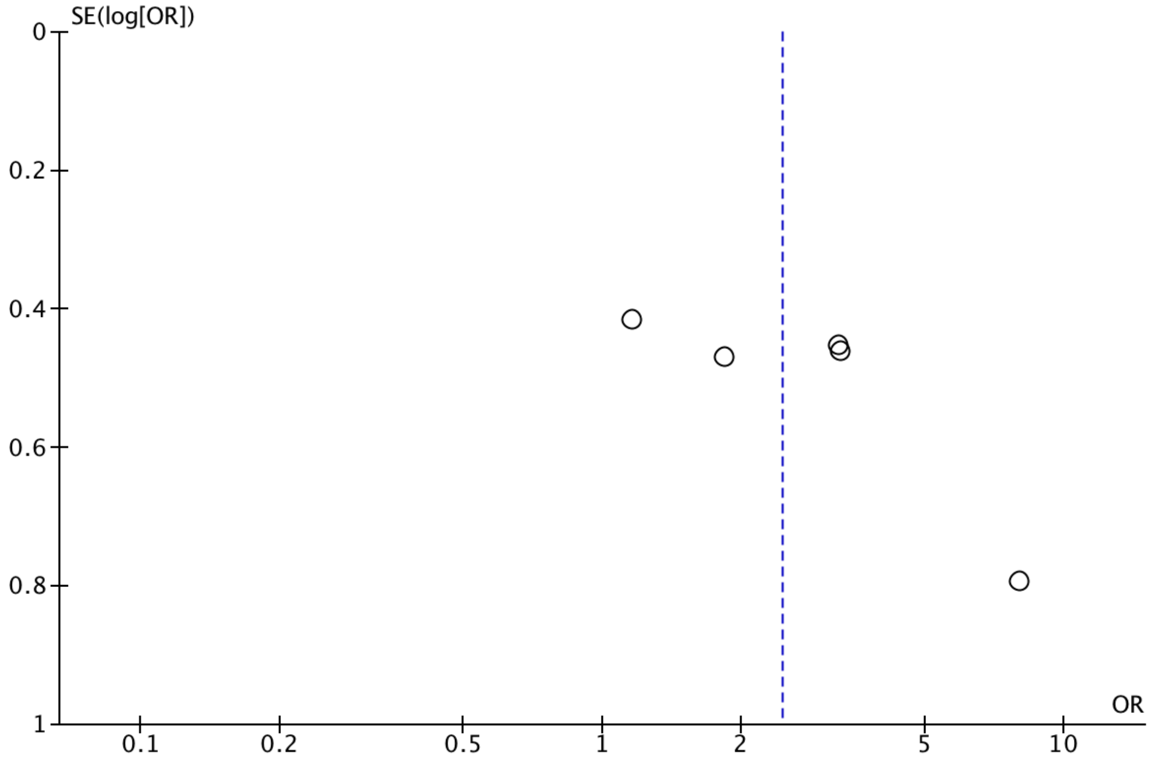

Figure S1. Funnel plot of studies included in the quantitative analysis (meta-analysis)
