# Supplementary material for: Intervertebral Disc Disease of the Lumbar Spine in Health Personnel with Occupational Exposure to Patient Handling—A Systematic Literature Review and Meta-Analysis
Source: Int J Environ Res Public Health. 2020 Jul 4;17(13):4832. doi: 10.3390/ijerph17134832 (PMC7370072; doi:10.3390/ijerph17134832)
Supplement: Supplementary file 1 [file ijerph-17-04832-s001.zip › Supplementary file_2.pdf]

Supplementary file 2 – Search terms and strategy used for Scopus, Pubmed, Web of Science, CINAHL databases

Table S2. Literature search in Scopus (10.01.2020)

No.	Search Terms	hits
#1	(moving lifting patients) OR (patient handling) OR (patient transfer)	128232
#2	therapist OR physiotherapist	91570
#3	(Health Personnel) OR nurse OR (care worker)	775928
#4	Occupation OR (Allied Health) OR (Work Related) OR (Working Environment) OR (Occupational Exposure)	778222
#5	**#1 OR #2 OR #3 OR #4**	**1681474**
#6	(Disc OR Disk) AND (Degeneration OR Herniation OR Disease OR Prolapse OR Protrusion OR Injury OR Displacement OR Disorder OR Herniated OR Bulging OR Degenerative) OR Spondylosis OR Osteochondrosis	92962
#7	(back pain)	100129
#8	Tomography OR (magnetic resonance imaging) OR (magnetic resonance imaging) OR MRI OR CT	1956177
#9	**#7 AND #8**	**23704**
#10	**#6 OR #9**	**111183**
#11	**#5 AND #10**	**2630**
#12	**#11 AND** (LIMIT-TO (SUBJAREA, "MEDI") OR LIMIT-TO (SUBJAREA, "HEAL") OR LIMIT-TO (SUBJAREA, "NEUR") OR LIMIT-TO (SUBJAREA, "NURS") OR LIMIT-TO (SUBJAREA, "MULT") OR LIMIT-TO (SUBJAREA, "Undefined")) AND (LIMIT-TO (EXACTKEYWORD, "Human")) AND (LIMIT-TO (DOCTYPE, "ar") OR LIMIT-TO (DOCTYPE, "re"))	**1937**

Table S3. Literature search in MEDLINE via PUBMED (10.01.2020)

No.	Search Terms Pubmed	hits
#1	"moving and lifting patients"[mh] OR ("moving"[All Fields] AND "lifting"[All Fields] AND "patients"[All Fields]) OR "moving and lifting patients"[All Fields] OR ("patient"[All Fields] AND "handling"[All Fields]) OR "patient handling"[All Fields] OR "patient transfer"[mh] OR ("patient"[All Fields] AND "transfer"[All Fields]) OR "patient transfer"[All Fields]	45115
#2	therapist[All Fields] OR "physical therapists"[mh] OR ("physical"[All Fields] AND "therapist"[All Fields]) OR "physical therapist"[All Fields] OR "physiotherapist"[All Fields] OR (occupational[All Fields] AND therapist[All Fields])	22834
#3	"Health Personnel"[mh] OR "Health Personnel"[All Fields] OR "nurse"[All Fields] OR "nurses"[All Fields] OR (care[All Fields] AND worker[All Fields])	813915
#4	Occupations[mh] OR Allied Health Occupations[mh] OR "Work-Related"[All Fields] OR „Work Related“[All Fields] OR "Working Environment"[All Fields] OR Occupational Exposure[mh] OR "Occupational Exposure"[All Fields] OR (Occupational[All Fields] AND Exposure[All Fields]) OR (Physical[All Fields] AND Load[All Fields])	233603
#5	**#1 OR #2 OR #3 OR #4**	**1075701**
#6	"Intervertebral Disc Degeneration"[mh] OR ((„Disc“[All Fields] OR „Disk“[All Fields]) AND „Degeneration“ [All Fields]) OR ((„Disc“[All Fields] OR „Disk“[All Fields]) AND („Herniation“ [All Fields] OR „Herniations“ [All Fields] OR „Disease“[All Fields] OR „Diseases“[All Fields] OR „Prolapse“ [All Fields] OR „Prolapses“ [All Fields] OR „Protrusion“[All Fields] OR „Protrusions“[All Fields] OR „Injury“[All Fields] OR „Injuries“[All Fields] OR „displacement“[All Fields] OR „disorder“[All Fields] OR „disorders“[All Fields] OR „Herniated“[All Fields] OR „Bulging“[All Fields] OR „Degenerative“[All Fields])) OR „Spondylosis“[mh] OR „Spondylosis“[All Fields] OR „Osteochondrosis"[mh] OR "Osteochondrosis"[All Fields] OR „Spondylarthrosis“[All Fields]	67844
#7	"back pain"[mh] OR ("back"[All Fields] AND "pain"[All Fields]) OR "back pain"[All Fields]	70254
#8	"Tomography"[mh] OR “Tomography”[All Fields] OR ("magnetic"[All Fields] AND "resonance"[All Fields] AND "imaging"[All Fields]) OR “magnetic resonance imaging”[All Fields] OR “MRI”[All Fields] OR “CT”[All Fields]	1327136
#9	**#7 AND #8**	**13455**
#10	**#6 OR #9**	**77987**
#11	**#5 AND #10**	**1355**
#12	**#11 Filters: Abstract; Humans**	**1082**

Table S4. Literature search in Web of Science (10.01.2020)

No.	**Search Terms** Science Citation Index Expanded (SCI-EXPANDED)	hits
#1	TS=((moving AND lifting AND patients) OR (patient AND handling) OR (patient AND transfer))	88540
#2	TS=(therapist OR physiotherapist)	24529
#3	TS=((Health Personnel) OR nurse OR (care worker))	251651
#4	TS=(Occupation OR (Allied Health) OR (Work Related) OR (Working Environment) OR (Occupational Exposure))	444103
#5	**#1 OR #2 OR #3 OR #4**	**775378**
#6	TS=((disc OR disk) AND (Degeneration OR Herniation OR Disease OR Prolapse OR Protrusion OR Injury OR Displacement OR Disorder OR Herniated OR Bulging OR Degenerative) OR Spondylosis OR Osteochondrosis)	**49037**
#7	TS=back pain	63771
#8	TS=(Tomography OR (magnetic resonance imaging) OR (magnetic resonance imaging) OR MRI OR CT)	1016366
#9	**#7 AND #8**	**8666**
#10	**#6 OR #9**	**55117**
#11	**#5 AND #10**	**1540**
#12	**#11 Refined by: DOCUMENT TYPES: (ARTICLE OR REVIEW) AND WEB OF SCIENCE CATEGORIES: (CLINICAL NEUROLOGY OR ORTHOPEDICS OR CRITICAL CARE MEDICINE OR SURGERY OR MECHANICS OR PUBLIC ENVIRONMENTAL OCCUPATIONAL HEALTH OR NURSING OR REHABILITATION OR SPORT SCIENCES OR RADIOLOGY NUCLEAR MEDICINE MEDICAL IMAGING OR OR NEUROSCIENCES OR EMERGENCY MEDICINE OR MEDICINE RESEARCH EXPERIMENTAL OR HEALTH CARE SCIENCES SERVICES OR HEALTH POLICY SERVICES OR ERGONOMICS OR PHYSIOLOGY OR MULTIDISCIPLINARY SCIENCES OR DENTISTRY ORAL SURGERY MEDICINE OR PRIMARY HEALTH CARE OR NEUROIMAGING OR ENVIRONMENTAL SCIENCES)**	**1046**

Table S5. Literature search in CINAHL (10.01.2020)

No.	Search Terms CINAHL (10.01.2020)<br>Expanders - Apply equivalent subjects<br>Search modes - Boolean/Phrase	hits
#1	(mh moving and lifting patients) OR (TX moving AND TX lifting AND TX patients) OR (mh patient handling+ OR TX patient AND TX handling) OR (mh patient transfer) OR (TX patient AND TX transfer) OR (TX patient transfer)	29784
#2	TX therapist OR (TX physical therapist) OR TX physiotherapist OR (TX occupational therapist)	75490
#3	(TX health professional) OR (TX health personnel) OR nurse OR TX nursing OR (TX health care worker)	1594027
#4	TX occupation OR (TX Allied Health Occupations) OR (TX work related) OR (TX Working Environment) OR (TX Occupational Exposure) OR (TX Physical Load)	56875
#5	**(S1 OR S2 OR S3 OR S4)**	**1706488**
#6	(TX intervertebral disc degeneration) OR (TX intervertebral disk degeneration) OR (TX Disc Herniation) OR (TX Disk Herniation) (TX disc disease) OR (TX disk disease) OR (TX disc prolapse) OR (TX disk prolapse) OR (TX disc protrusion) OR (TX disk protrusion) OR (TX disc injury) OR (TX disk injury) OR (TX disc displacement, intervertebral) OR (TX disk displacement, intervertebral) OR (TX disc disorder) OR (TX disk disorder) OR (TX Disc Herniated) OR (TX Disk Herniated) OR (TX Disc Bulging) OR (TX Disk Bulging) OR (TX Disc Degenerative) OR (TX Disk Degenerative) OR TX spondylosis OR TX Spondylarthrosis	7736
#7	TX back pain	36220
#8	TX Tomography OR TX (magnetic resonance imaging) OR MRI OR CT	268783
#9	**#7 AND #8**	**4131**
#10	**#6 OR #9**	**11112**
#11	**#5 AND #10**	**960**
#12	**#11 Limiters - Abstract Available; Human; Age Groups: Adolescent: 13-18 years, Adult: 19-44 years, Middle Aged: 45-64 years, Aged: 65+ years, Aged, 80 and over**	**196**
