# Supplementary material for: Intervertebral Disc Disease of the Lumbar Spine in Health Personnel with Occupational Exposure to Patient Handling—A Systematic Literature Review and Meta-Analysis
Source: Int J Environ Res Public Health. 2020 Jul 4;17(13):4832. doi: 10.3390/ijerph17134832 (PMC7370072; doi:10.3390/ijerph17134832)
Supplement: Supplementary file 1 [file ijerph-17-04832-s001.zip › Supplementary file_3.pdf]

Supplementary file 3: Newcastle-Ottawa quality assessment form for cohort studies, case-control studies and cross-sectional studies
Note: A study can be given a maximum of one star (+) for each numbered item within the Selection and Outcome categories. A maximum of two stars can be given for Comparability.

Selection
1) Representativeness of the exposed cohort
   a) Truly representative +
   b) Somewhat representative +
   c) Selected group
   d) No description of the derivation of the cohort
2) Selection of the non-exposed cohort
   a) Drawn from the same community as the exposed cohort +
   b) Drawn from a different source
   c) No description of the derivation of the non-exposed cohort
   d) comparison within the group
3) Ascertainment of exposure
   a) Secure record (e.g., surgical record) +
   b) Structured interview +
   c) Written self-report
   d) No description
   e) Other
4) Demonstration that outcome of interest was not present at start of study
   a) Yes +
   b) No

Comparability
1) Comparability of cohorts on the basis of the design or analysis controlled for confounders
   a) The study controls for age and sex ++
   b) The study controls for age or sex +
   c) The study controls other factors, but not age or sex: ________________________
   d) Cohorts are not comparable on the basis of the design or analysis controlled for confounders

Outcome
1) Assessment of outcome
   a) Independent blind assessment +
   b) Record linkage +
   c) Self report
   d) No description
   e) Other
2) Was follow-up long enough for outcomes to occur
   a) Yes +
   b) No
3) Were objective standard criteria used for measurement of the condition
   a) MRI +
   b) ICD
4) Adequacy of follow-up of cohorts
   a) Complete follow up- all subject accounted for +
   b) Subjects lost to follow up unlikely to introduce bias-number lost less than or equal to 20% or description of those lost suggested no different from those followed. +
   c) Follow up rate less than 80% and no description of those lost
   d) No statement

Thresholds for converting the Newcastle-Ottawa scales to AHRQ standards (good, fair, and poor):

Good quality: 3 or 4 stars in selection domain **AND** 1 or 2 stars in comparability domain **AND** 3 or 4 stars in outcome/exposure domain
Fair quality: 2 stars in selection domain **AND** 1 or 2 stars in comparability domain **AND** 2 or 3 stars in outcome/exposure domain
Poor quality: 0 or 1 star in selection domain **OR** 0 stars in comparability domain **OR** 0 or 1 stars in outcome/exposure domain

Table S6. Quality assessment of included cohort studies

**Author, year**	**Selection (Maximum 4 +)**	**Comparability (Maximum 2 +)**	**Outcome (Maximum 3 +)**	**Study quality***
Heliovaara et al. 1987	1b; 2a; 3b; 4b (+++)	1a (++)	1b; 2a; 3b; 4d (++)	++
Chung et al. 2013	1a; 2a; 3a; 4a (++++)	1a (++)	1b; 2a; 3b; 4d (+++)	+++
Makino et al. 2017	1b; 2d; 3a; 4b (++)	1b (+)	1a; 2a; 3a; 4c (+++)	++

* AHRQ standard: good (+++), fair (++) and poor (+)

NEWCASTLE - OTTAWA QUALITY ASSESSMENT SCALE (adapted for cross sectional studies)
This scale has been adapted from the Newcastle-Ottawa Quality Assessment Scale for cohort studies to perform a quality assessment of cross-sectional studies for the systematic review, "Are Healthcare Workers' Intentions to Vaccinate Related to their Knowledge, Beliefs and Attitudes? A Systematic Review".

Selection (Maximum 4 +)
1) Representativeness of the sample:
   a) Truly representative of the average in the target population. + (all subjects or random sampling)
   b) Somewhat representative of the average in the target population. + (non-random sampling)
   c) Selected group of users.
   d) No description of the sampling strategy.
2) Sample size:
   a) Justified and satisfactory. +
   b) Not justified.
3) Non-respondents:
   a) Comparability between respondents and non-respondents characteristics is established, and the response rate is satisfactory. +
   b) The response rate is unsatisfactory, or the comparability between respondents and non-respondents is unsatisfactory.
   c) No description of the response rate or the characteristics of the responders and the non-responders.
4) Ascertainment of the exposure (risk factor):
   a) Validated measurement tool. ++
   b) Non-validated measurement tool, but the tool is available or described. +
   c) No description of the measurement tool.

Comparability
1) The subjects in different outcome groups are comparable, based on the study design or analysis. Confounding factors are controlled.
   a) The study controls for the age and sex. ++
   b) The study controls for age or sex. +
   c) The study controls for confounding, but not age or sex.
   d) No confounder control in analyses and no matching

Outcome
1) Assessment of the outcome:
   a) Independent blind assessment. ++
   b) Record linkage. ++
   c) Self report. +
   d) No description.
2) Statistical test:
   a) The statistical test used to analyze the data is clearly described and appropriate, and the measurement of the association is presented, including confidence intervals and the probability level (p value). +
   b) The statistical test is not appropriate, not described or incomplete.
3) Were objective standard criteria used for measurement of the condition
   a) MRI +
   b) ICD

Thresholds for converting the Newcastle-Ottawa scales to AHRQ standards (good, fair, and poor):

Good quality: 3 or 4 stars in selection domain **AND** 1 or 2 stars in comparability domain **AND** 3 or 4 stars in outcome/exposure domain
Fair quality: 2 stars in selection domain **AND** 1 or 2 stars in comparability domain **AND** 2 or 3 stars in outcome/exposure domain
Poor quality: 0 or 1 star in selection domain **OR** 0 stars in comparability domain **OR** 0 or 1 stars in outcome/exposure domain

Table S7. Quality assessment of included cross-sectional studies

Author, year	Selection (Maximum 4 +)	Comparability (Maximum 2 +)	Outcome (Maximum 3 +)	Study quality*
Hartwig et al. 1997	1c; 2b; 3c; 4c (-)	1d (-)	1d; 2b; 3a (+)	+
Savage et al. 1997	1d; 2b; 3c; 4a (++)	1c (-)	1a; 2b; 3a (+++)	+
D'Agostin et al. 2017	1a; 2a; 3c; 4a (++++)	1d (-)	1b; 2c; 3a (+++)	+

* AHRQ standard: good (+++), fair (++) and poor (+)

Newcastle-Ottawa Quality Assessment Form for Case-Control Studies
Note: A study can be awarded a maximum of one star (+) for each numbered item within the Selection and Exposure categories. A maximum of two stars can be given for Comparability.

Selection

1) Is the case definition adequate?
   - a) yes, with independent validation +
   - b) yes, e.g., record linkage or based on self-reports
   - c) other data
   - d) no description
2) Representativeness of the cases
   - a) consecutive or obviously representative series of cases +
   - b) potential for selection biases or not stated
3) Selection of Controls
   - a) community controls +
   - b) hospital controls
   - c) no description
4) Definition of Controls
   - a) no history of disease (endpoint) +
   - b) no description of source

Comparability

1) Comparability of cases and controls on the basis of the design or analysis
   - a) The study controls for age and sex ++
   - b) The study controls for age or sex +
   - c) The study controls other factors, but not age or sex: ________________________
   - d) Cohorts are not comparable on the basis of the design or analysis controlled for confounders

Exposure

1) Ascertainment of exposure
   - a) secure record (e.g. surgical records) +
   - b) structured interview where blind to case/control status +
   - c) interview not blinded to case/control status)
   - d) written self-report or medical record
2) Same method of ascertainment for cases and controls
   - a) Yes +
   - b) No
3) Non-Response rate
   - a) same rate for both groups +
   - b) non respondents described
   - c) rate different and no designation

Thresholds for converting the Newcastle-Ottawa scales to AHRQ standards (good, fair, and poor):

Good quality: 3 or 4 stars in selection domain **AND** 1 or 2 stars in comparability domain **AND** 3 or 4 stars in outcome/exposure domain
Fair quality: 2 stars in selection domain **AND** 1 or 2 stars in comparability domain **AND** 2 or 3 stars in outcome/exposure domain
Poor quality: 0 or 1 star in selection domain **OR** 0 stars in comparability domain **OR** 0 or 1 stars in outcome/exposure domain

Table S8. Quality assessment of included case-control studies

**Author, year**	**Selection (Maximum 4 +)**	**Comparability (Maximum 2 +)**	**Exposure (Maximum 3 +)**	**Study quality***
Michaelis et al. 2001	1a; 2b; 3a; 4a (+++)	1a (++)	1d; 2a; 3a (++)	+++

* AHRQ standard: good (+++), fair (++) and poor (+)

References

Modesti PA, Reboldi G, Cappuccio FP, et al. Panethnic Differences in Blood Pressure in Europe: A Systematic Review and Meta-Analysis. PLoS One. 2016;11(1):e0147601. Published 2016 Jan 25. doi:10.1371/journal.pone.0147601

Wells, G.; Shea, B.; O'Connell, D.; Peterson, j.; Welch, V.; Losos, M.; Tugwell, P. The Newcastle–Ottawa Scale (NOS) for Assessing the Quality of Non-Randomized Studies in Meta-Analysis. 2000.
