# Supplementary material for: Intervertebral Disc Disease of the Lumbar Spine in Health Personnel with Occupational Exposure to Patient Handling—A Systematic Literature Review and Meta-Analysis
Source: Int J Environ Res Public Health. 2020 Jul 4;17(13):4832. doi: 10.3390/ijerph17134832 (PMC7370072; doi:10.3390/ijerph17134832)
Supplement: Supplementary file 1 [file ijerph-17-04832-s001.zip › Supplementary file_1.pdf]

Supplementary file 1 – A proposed reporting checklist for authors, editors and reviewers of meta-analyses of observational studies in epidemiology (MOOSE)

Table S1. MOOSE (Meta-analyses Of Observational Studies in Epidemiology) Checklist

Checklist	Yes/no/ n.a.
**Reporting of background**	
1. Problem definition	**Yes**
2. Hypothesis statement	**Yes**
3. Description of study outcome	**Yes**
4. Type of exposure or intervention used	**Yes**
5. Type of study design used	**Yes**
6. Study population	**Yes**
**Reporting of search strategy should include**	
7. Qualification of searchers (e.g. librarians)	**Yes**
8. Search strategy, including time period included in the synthesis and keywords	**Yes**
9. Effort to include all available studies, including contact with authors	**Yes**
10. Databases and registries searched	**Yes**
11. Search software used, name, version, including special features used (e.g. explosion)	**Yes**
12. Use of hand searching (e.g. reference lists of obtained studies)	**Yes**
13. List of citations located and those excluded, including justification	**Yes**
14. Method of addressing articles published in languages other than English	**Yes**
15. Methods of handling abstracts and unpublished studies	**No**[1]
16. Description of any contact with authors	**Yes**
**Reporting of methods should include**	
17. Description of relevance or appropriateness of studies assembled for assessing the hypothesis to be tested	**Yes**
18. Rationale for the selection and coding of data	**Yes**
19. Documentation of how data were classified and coded (e.g. multiple raters, blinding and interrater reliability)	**Yes**
20. Assessment of confounding (e.g. comparability of cases and controls in studies where appropriate)	**Yes**
21. Assessment of study quality, including blinding of quality assessors, stratification or regression possible predictors of study results	**Yes**
22. Assessment of heterogeneity	**Yes**
23. Description of statistical methods (e.g. complete description of fixed or random effect models, justification of whether the chosen models account for predictors of study results, dose-response models, or cumulative meta- analysis) in sufficient detail to be replicated	**Yes**
24. Provision of appropriate tables and graphics	**Yes**
**Reporting of results should include**	
25. Graphic summarising individual study estimates and overall estimate	**Yes**
26. Table giving descriptive information for each study included	**Yes**
27. Results of sensitivity testing (e.g. subgroup analysis)	**Yes**
28. Indication of statistical uncertainty of findings	**Yes**
**Reporting of discussion should include**	
29. Quantitative assessment of bias (e.g. publication bias)	**Yes**
30. Justification for exclusion (e.g. exclusion of non-English-language yes citations)	**Yes**
31. Assessment of quality of the included studies	**Yes**

Reporting of conclusions should include

32. Consideration of alternative explanations for the observed results	**Yes**
33. Generalization of the conclusions (i.e. appropriate for the data presented yes and within the domain of the literature review)	**Yes**
34. Guidelines for future research	**Yes**
35. Disclosure of funding source	**Yes**

[1]We did not attempt to identify unpublished data.
